# Supplementary material for: The human posterior parietal cortices orthogonalize the representation of different streams of information concurrently coded in visual working memory
Source: PLoS Biol. 2024 Nov 21;22(11):e3002915. doi: 10.1371/journal.pbio.3002915 (PMC11620661; doi:10.1371/journal.pbio.3002915)
Supplement: S1 Fig — (A) Experiment 1 behavioral VWM change detection performance. (B) Experiment 2 behavioral VWM change detection performance. Error bars indicate SE. Data are available from S1 Data and at osf.io/8rbkh/. (PDF) [file pbio.3002915.s001.pdf]

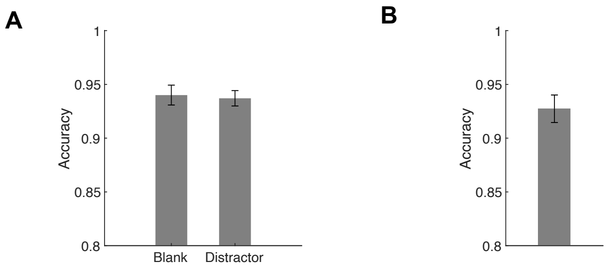

<u>S1 Fig.</u> Behavioral performance. **A.** Experiment 1 behavioral VWM change detection performance. **B.** Experiment 2 behavioral VWM change detection performance. Error bars indicate s.e. Data are available from the supplemental data file and at osf.io/8rbkh/.
