# Supplementary material for: The human posterior parietal cortices orthogonalize the representation of different streams of information concurrently coded in visual working memory
Source: PLoS Biol. 2024 Nov 21;22(11):e3002915. doi: 10.1371/journal.pbio.3002915 (PMC11620661; doi:10.1371/journal.pbio.3002915)
Supplement: S2 Fig — (A) Average number of vertices in each ROI. Error bars indicate SE. (B) Time courses of beta weights for trials with and without distractors averaged over all the vertices within each ROI. In each ROI plot, the light gray vertical bars mark the stimulus presentation time during the encoding and probe periods, the medium gray vertical bars mark the fMRI decoding period during VWM delay, and the dark gray horizontal bar marks the distractor presentation time. See Materials and methods for more details. The lighter-colored ribbons around the plot lines represent SE. Data are available from S1 Data and at osf.io/8rbkh/. (PDF) [file pbio.3002915.s002.pdf]

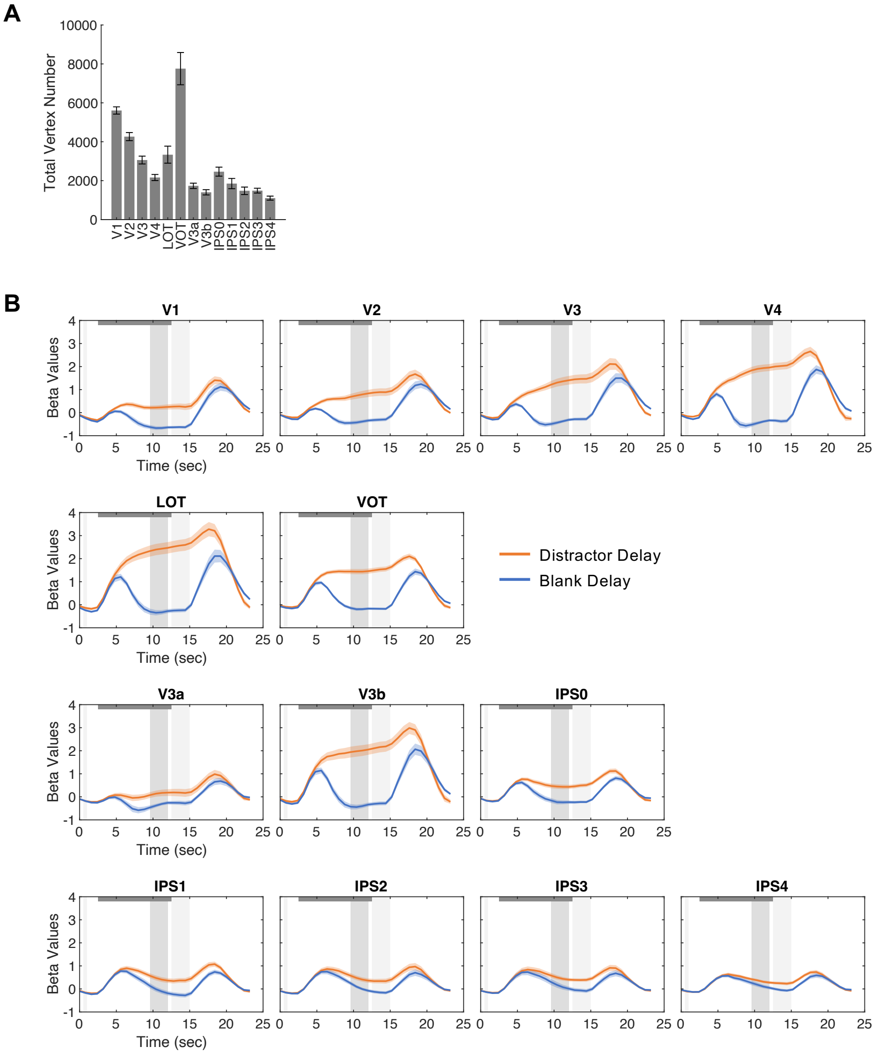

S2 Fig. Experiment 1 vertex numbers and response time courses of each ROI. **A.** Average number of vertices in each ROI. Error bars indicate s.e. **B.** Time courses of beta weights for trials with and without distractors averaged over all the vertices within each ROI. In each ROI plot, the light gray vertical bars mark the stimulus presentation time during the encoding and probe periods, the medium gray vertical bars mark the fMRI decoding period during VWM delay, and the dark gray horizontal bar marks the distractor presentation time. See Methods for more details. The lighter-colored ribbons around the plot lines represent s.e**.** Data are available from the supplemental data file and at osf.io/8rbkh/.
