# Supplementary material for: The human posterior parietal cortices orthogonalize the representation of different streams of information concurrently coded in visual working memory
Source: PLoS Biol. 2024 Nov 21;22(11):e3002915. doi: 10.1371/journal.pbio.3002915 (PMC11620661; doi:10.1371/journal.pbio.3002915)
Supplement: S3 Fig — (A and B) Target object decoding accuracy during VWM delay for trials with and without distractors for all the ROIs and for the 3 ROI sectors, respectively. The colored symbols above the bars mark the decoding significance of each bar compared to chance (.5). The black symbols mark the significance in decoding difference between trials with and without distractors. Error bars indicate SE. * p < .05, ** .001 < p < .01, *** p < .001. (C) Target and distractor decoding across time. In each ROI plot, the light gray vertical bars mark the stimulus presentation time during the encoding and probe periods, the medium gray vertical bars mark the fMRI decoding period for VWM delay, and the dark gray horizontal bar marks the distractor presentation time. See Materials and methods for more details. The horizontal dashed line indicates chance level decoding. The lighter-colored ribbons around the plot lines represent SE. Data are available from S1 Data and at osf.io/8rbkh/. (PDF) [file pbio.3002915.s003.pdf]

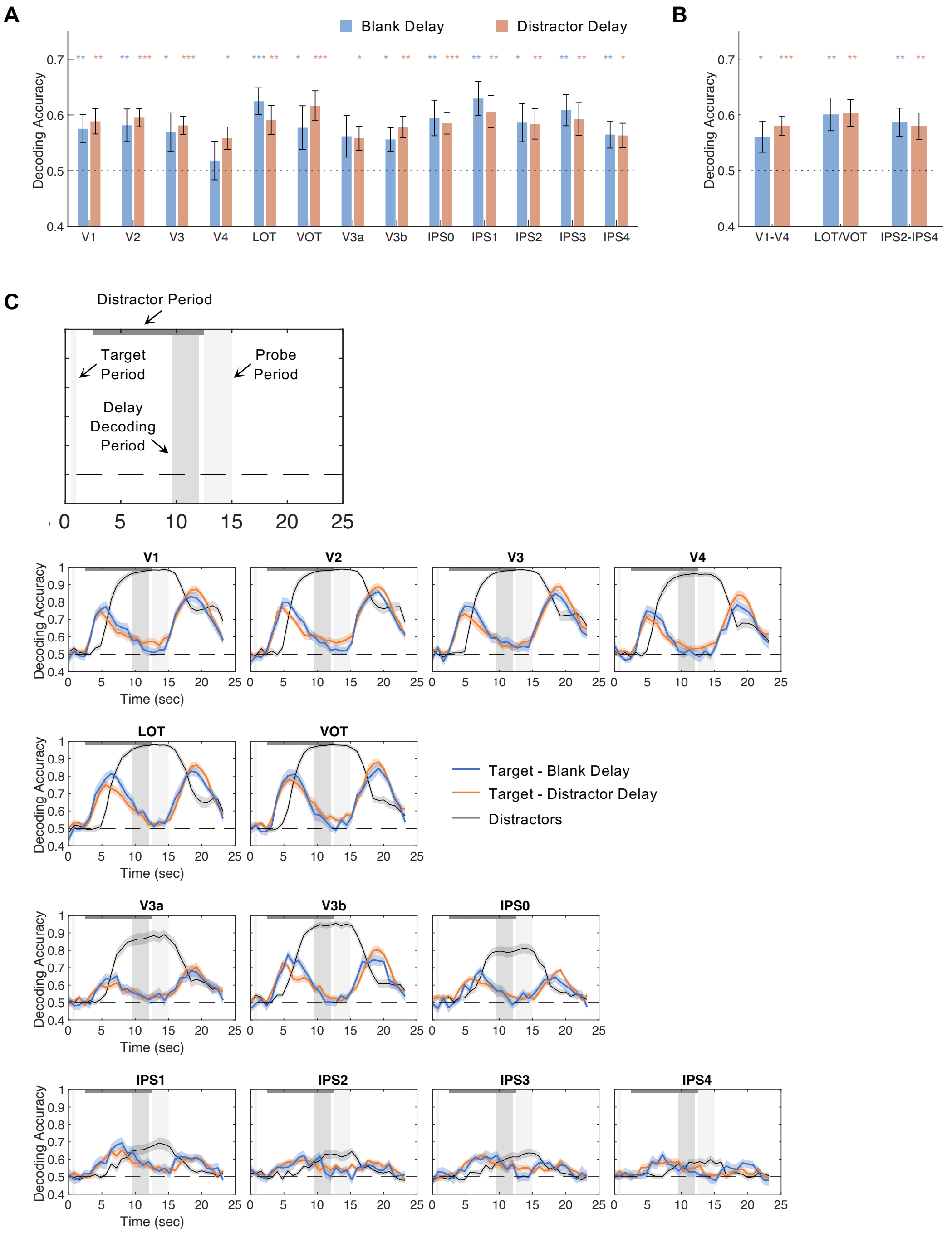

<u>S3 Fig.</u> Experiment 1 target and distractor decoding for trials with and without distractors. **A** and **B.** Target object decoding accuracy during VWM delay for trials with and without distractors for all the ROIs and for the three ROI sectors, respectively. The colored symbols above the bars mark the decoding significance of each bar compared to chance (.5). The black symbols mark the significance in decoding difference between trials with and without distractors. Error bars indicate s.e. * $p < .05$, ** $.001 < p < .01$, *** $p < .001$. **C.** Target and distractor decoding across time. In each ROI plot, the light gray vertical bars mark the stimulus presentation time during the encoding and probe periods, the medium gray vertical bars mark the fMRI decoding period for VWM delay, and the dark gray horizontal bar marks the distractor presentation time. See Methods for more details. The horizontal dashed line indicates chance level decoding. The lighter-colored ribbons around the plot lines represent s.e**.** Data are available from the supplemental data file and at osf.io/8rbkh/.
