# Supplementary material for: The human posterior parietal cortices orthogonalize the representation of different streams of information concurrently coded in visual working memory
Source: PLoS Biol. 2024 Nov 21;22(11):e3002915. doi: 10.1371/journal.pbio.3002915 (PMC11620661; doi:10.1371/journal.pbio.3002915)
Supplement: S4 Fig — Blue lines depict untransformed decoding accuracy as a function of the underlying signal strength. Orange lines depict probit-transformed decoding accuracy as a function of the underlying signal strength. See S1 Supplementary Results for more details. (PDF) [file pbio.3002915.s004.pdf]

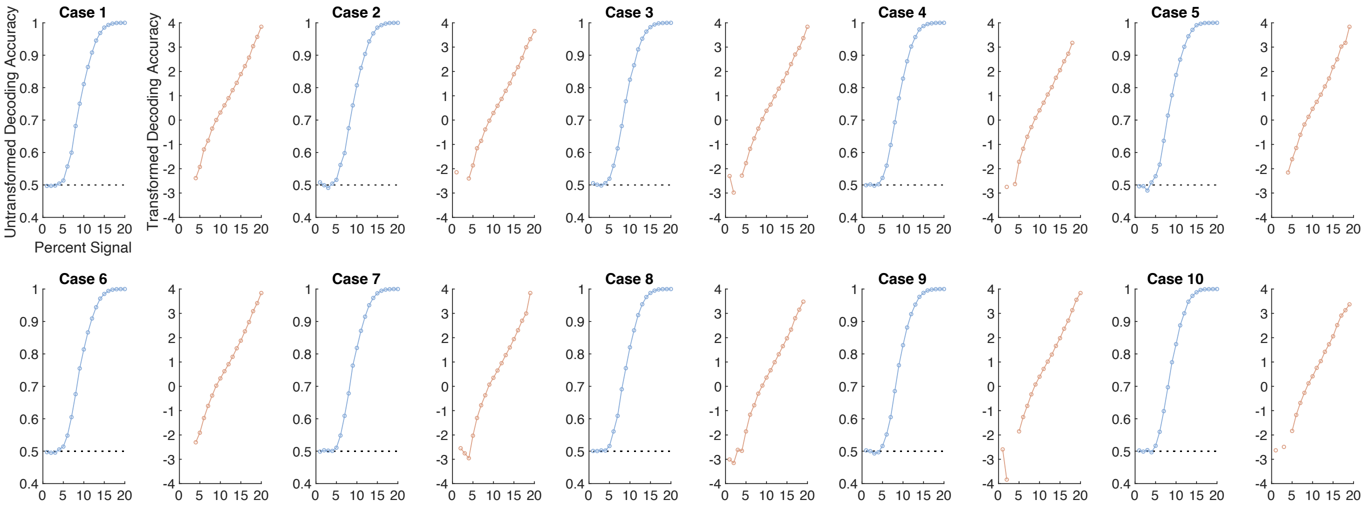

<u>S4 Fig.</u> Simulation results from 10 cases. Blue lines depict untransformed decoding accuracy as a function of the underlying signal strength. Orange lines depict probit-transformed decoding accuracy as a function of the underlying signal strength. See Supplementary Results for more details.
