# Supplementary material for: The human posterior parietal cortices orthogonalize the representation of different streams of information concurrently coded in visual working memory
Source: PLoS Biol. 2024 Nov 21;22(11):e3002915. doi: 10.1371/journal.pbio.3002915 (PMC11620661; doi:10.1371/journal.pbio.3002915)
Supplement: S5 Fig — (A) Within- and cross-decoding over time of targets across trials with different types of distractors. (B) Within- and cross-decoding over time of targets across trials with and without distractors. In each ROI plot, the light gray vertical bars mark the stimulus presentation time during the encoding and probe periods, the medium gray vertical bars mark the fMRI decoding period for VWM delay, and the dark gray horizontal bar marks the distractor presentation time. See Materials and methods for more details. The horizontal dashed line indicates chance level decoding. The lighter-colored ribbons around the plot lines represent SE. Data are available at osf.io/8rbkh/. (PDF) [file pbio.3002915.s005.pdf]

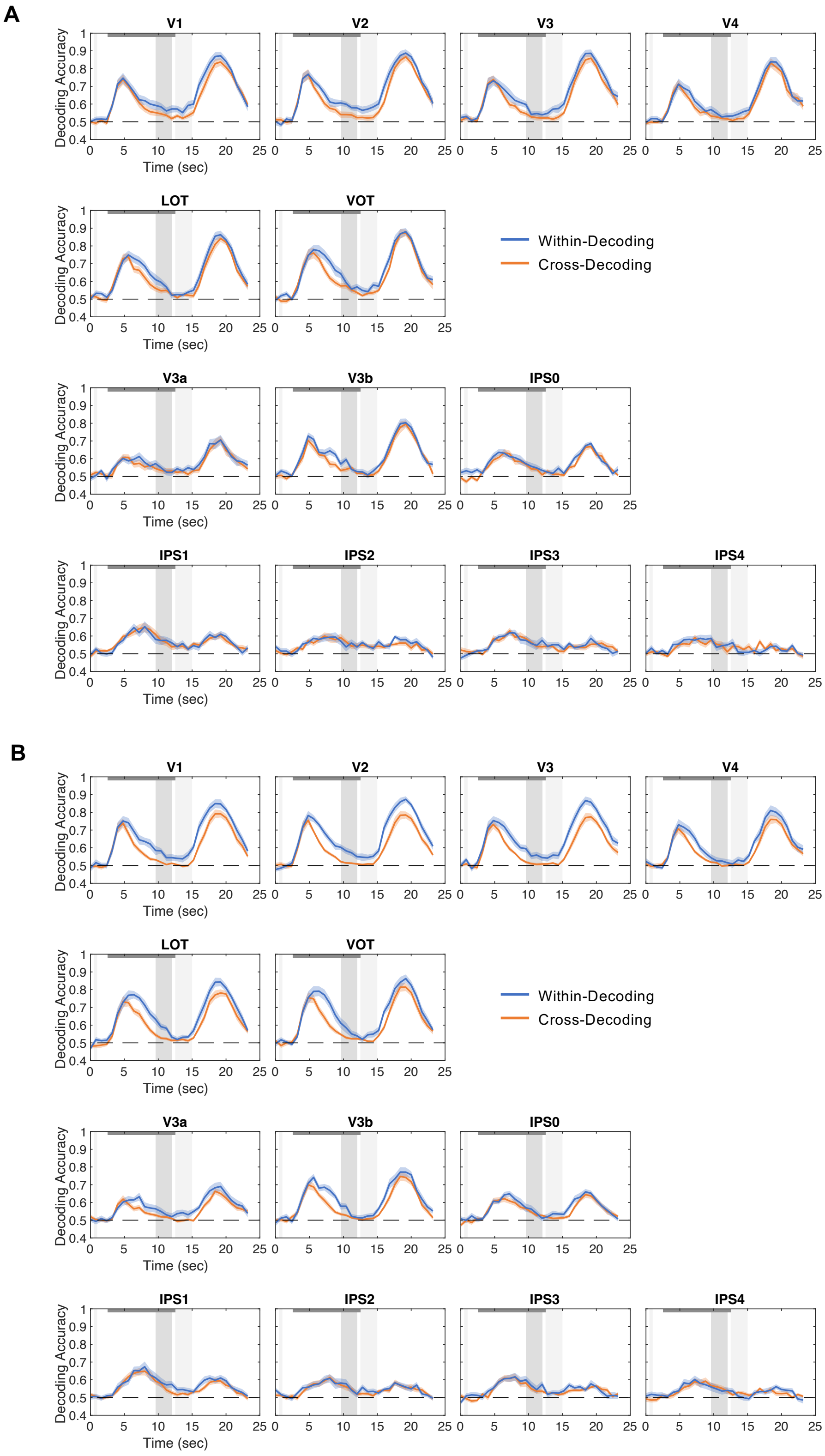

S5 Fig. Experiment 1 decoding time courses. **A.** Within- and cross-decoding over time of targets across trials with different types of distractors. **B.** Within- and cross-decoding over time of targets across trials with and without distractors. In each ROI plot, the light gray vertical bars mark the stimulus presentation time during the encoding and probe periods, the medium gray vertical bars mark the fMRI decoding period for VWM delay, and the dark gray horizontal bar marks the distractor presentation time. See Methods for more details. The horizontal dashed line indicates chance level decoding. The lighter-colored ribbons around the plot lines represent s.e**.** Data are available at osf.io/8rbkh/.
