# Supplementary material for: The human posterior parietal cortices orthogonalize the representation of different streams of information concurrently coded in visual working memory
Source: PLoS Biol. 2024 Nov 21;22(11):e3002915. doi: 10.1371/journal.pbio.3002915 (PMC11620661; doi:10.1371/journal.pbio.3002915)
Supplement: S6 Fig — (A). Representational space for targets and distractors with different target-distractor pairing. Each representational space geometry is an MDS projection of the group-averaged RDM of the 4 conditions included. Here, 2 types of targets (pink and purple) are paired with 2 types of distractors (black and grey lines). In a given trial, only 1 target (pink or purple) is shown with 1 type of distractors (black or grey line) (see Fig 1A). See main text for more details. (B) Target-target angles and (C) distractor-distractor angles. In each plot, angles calculated from the RDMs of the individual participants are shown on the left and those from the group RDMs are shown on the right. See main text for more details. Error bars indicate SE. Data are available from S1 Data and at osf.io/8rbkh/. (PDF) [file pbio.3002915.s006.pdf]

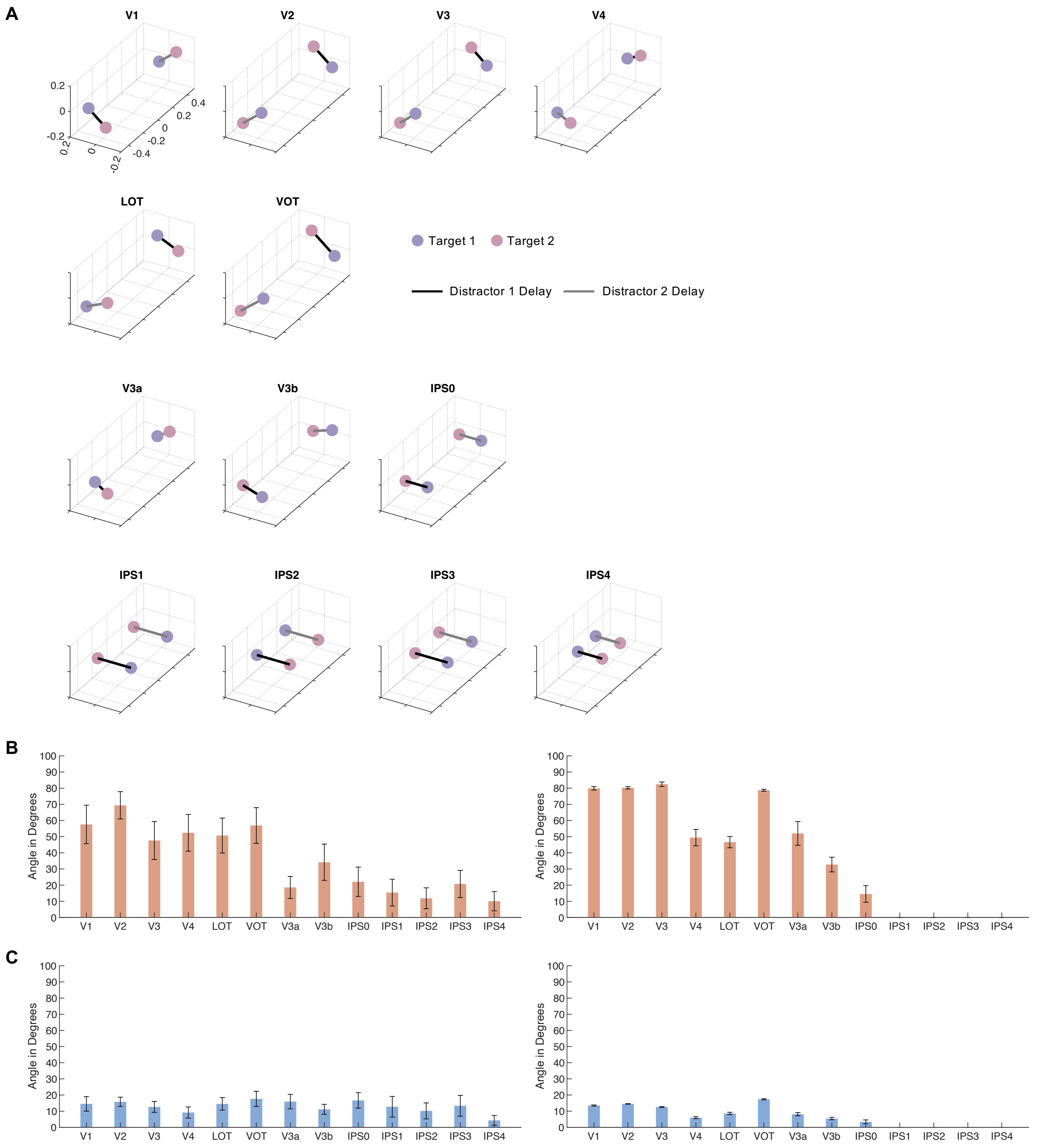

S6 Fig. Experiment 1 angles of target and distractor representations across trials with different types of distractors for each ROI. **A.** Representational space for targets and distractors with different target-distractor pairing. Each representational space geometry is an MDS projection of the group-averaged RDM of the four conditions included. Here two types of targets (pink and purple) are paired with two types of distractors (black and grey lines). In a given trial, only one target (pink or purple) is shown with one type of distractors (black or grey line) (see Figure 1A). See main text for more details. **B.** Target-target angles and **C.** Distractor-distractor angles. In each plot, angles calculated from the RDMs of the individual participants are shown on the left and those from the group RDMs are shown on the right. See main text for more details. Error bars indicate s.e. Data are available from the supplemental data file and at osf.io/8rbkh/.
