# Supplementary material for: The human posterior parietal cortices orthogonalize the representation of different streams of information concurrently coded in visual working memory
Source: PLoS Biol. 2024 Nov 21;22(11):e3002915. doi: 10.1371/journal.pbio.3002915 (PMC11620661; doi:10.1371/journal.pbio.3002915)
Supplement: S7 Fig — (A) Representational space for targets and distraction conditions, as in S6A Fig. (B) Target-target angles and (C) distraction condition angles, as in S6B and S6C Fig. In each plot, angles calculated from the RDMs of the individual participants are shown on the left and those from the group RDMs are shown on the right. Error bars indicate SE. Data are available from S1 Data and at osf.io/8rbkh/. (PDF) [file pbio.3002915.s007.pdf]

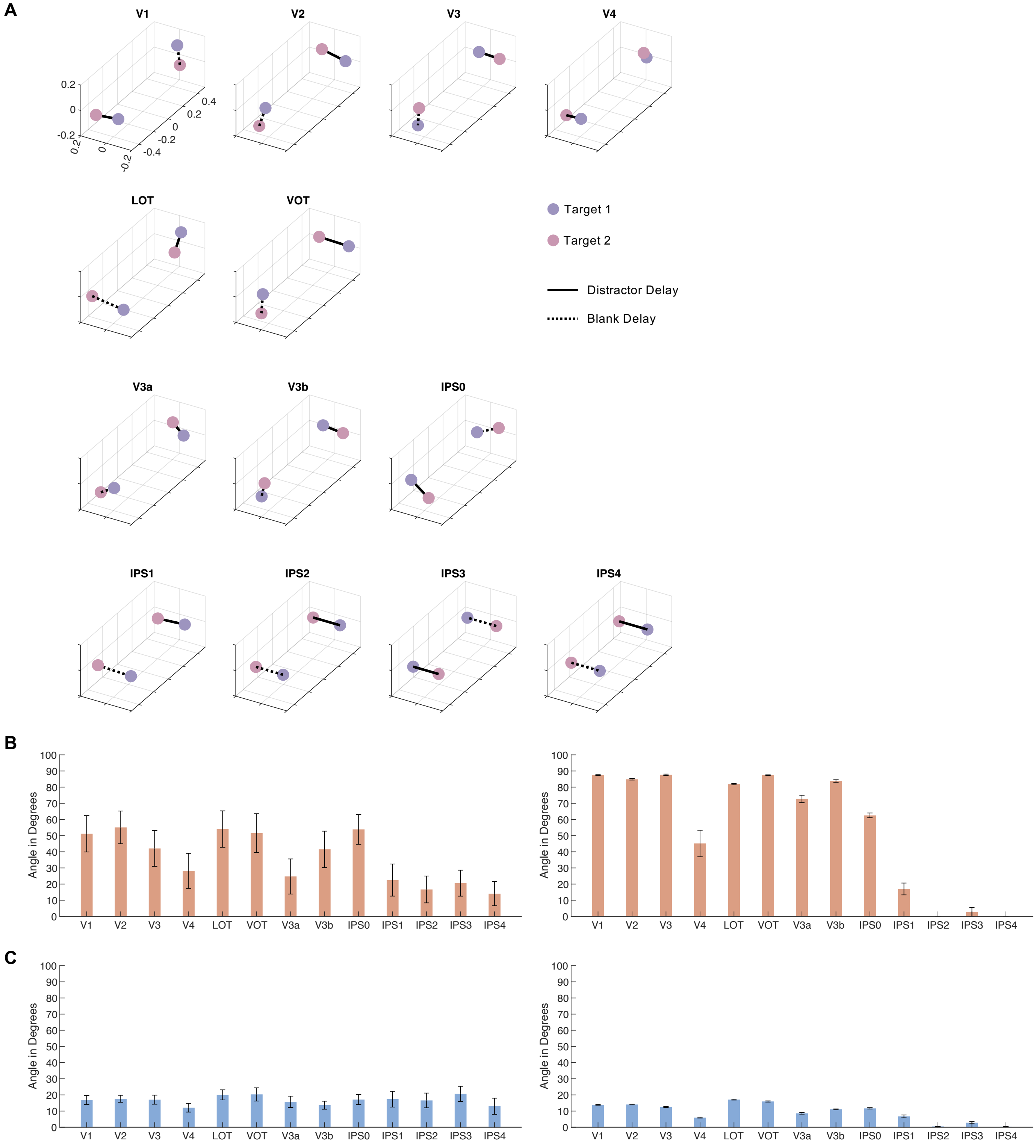

<u>S7 Fig.</u> Experiment 1 angles of target and distractor representations across trials with and without distractors for each ROI. **A.** Representational space for targets and distraction conditions, as in Supplementary Figure 6A. **B.** Target-target angles and **C.** Distraction condition angles, as in Supplementary Figures 6B and 6C. In each plot, angles calculated from the RDMs of the individual participants are shown on the left and those from the group RDMs are shown on the right. Error bars indicate s.e. Data are available from the supplemental data file and at osf.io/8rbkh/.
