# Supplementary material for: The human posterior parietal cortices orthogonalize the representation of different streams of information concurrently coded in visual working memory
Source: PLoS Biol. 2024 Nov 21;22(11):e3002915. doi: 10.1371/journal.pbio.3002915 (PMC11620661; doi:10.1371/journal.pbio.3002915)
Supplement: S9 Fig — (A) An illustration of the presentation order decoding. Here, a decoder is trained to decode trials containing the same 2 target objects but in different presentation orders. (B and C) Order decoding accuracy during VWM encoding and delay for all the ROIs and the 3 ROI sectors, respectively. The colored symbols above the bars mark the decoding significance of each bar compared to chance (.5). The black symbols mark the significance in decoding difference between the encoding and delay periods. The horizontal dashed line indicates chance level decoding. Error bars indicate SE. * p < .05, ** .001 < p < .01, *** p < .001. (D) Time courses of order decoding for all the ROIs. In each ROI plot, the light gray vertical bars mark the stimulus presentation time during the encoding and probe periods, and the medium gray vertical bars mark the fMRI decoding period for the VWM encoding and delay periods. See Materials and methods for more details. The horizontal dashed line indicates chance level decoding. The lighter-colored ribbons around the plot lines represent SE. Data are available from S1 Data and at osf.io/8rbkh/. (PDF) [file pbio.3002915.s009.pdf]

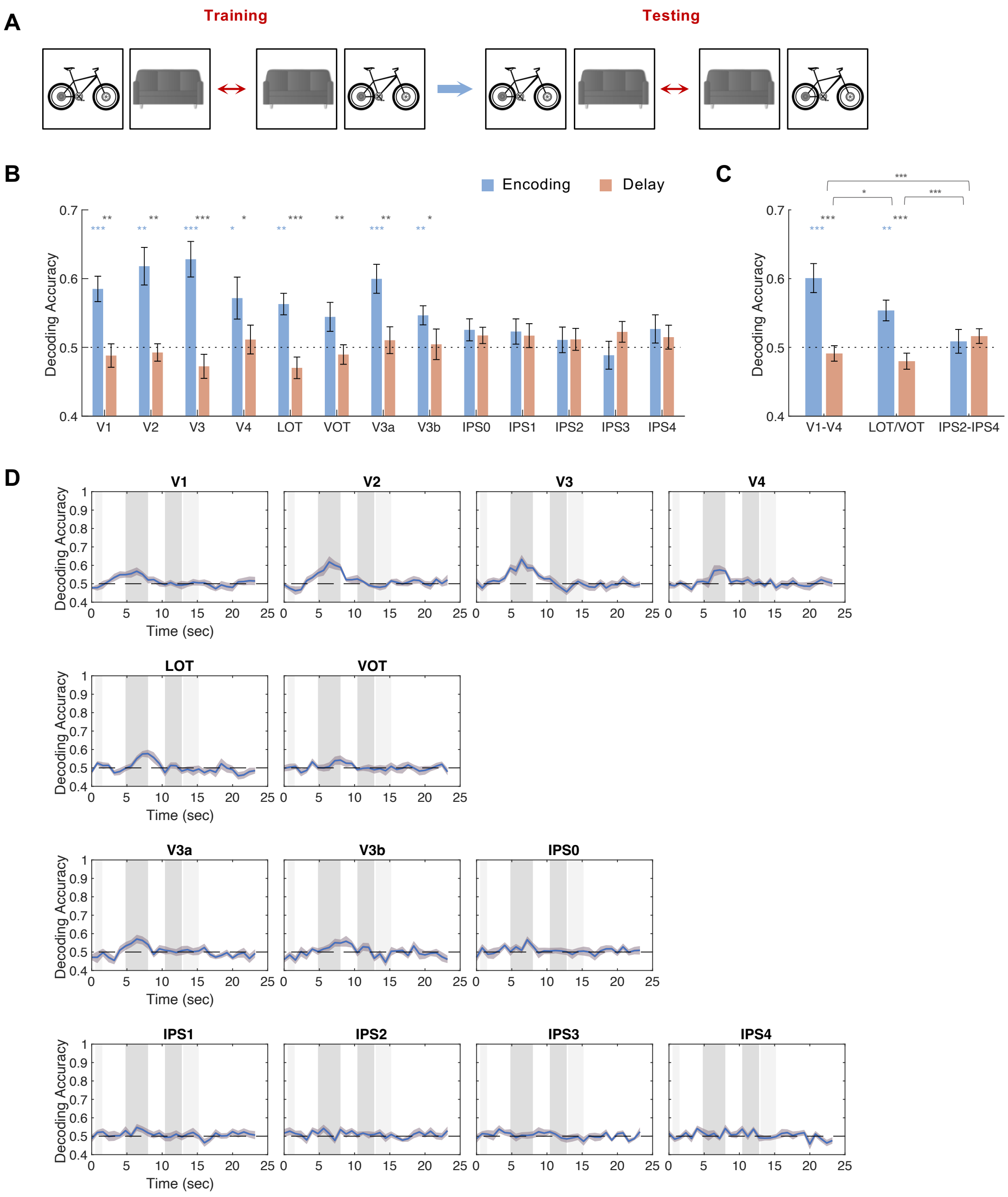

<u>S9 Fig.</u> Experiment 2 presentation order decoding. **A.** An illustration of the presentation order decoding. Here a decoder is trained to decode trials containing the same two target objects but in different presentation orders. **B** and **C.** Order decoding accuracy during VWM encoding and delay for all the ROIs and the three ROI sectors, respectively. The colored symbols above the bars mark the decoding significance of each bar compared to chance (.5). The black symbols mark the significance in decoding difference between the encoding and delay periods. The horizontal dashed line indicates chance level decoding. Error bars indicate s.e. * $p < .05$, ** $.001 < p < .01$, *** $p < .001$. **D.** Time courses of order decoding for all the ROIs. In each ROI plot, the light gray vertical bars mark the stimulus presentation time during the encoding and probe periods, and the medium gray vertical bars mark the fMRI decoding period for the VWM encoding and delay periods. See Methods for more details. The horizontal dashed line indicates chance level decoding. The lighter-colored ribbons around the plot lines represent s.e**.** Data are available from the supplemental data file and at osf.io/8rbkh/.
