# Supplementary material for: The human posterior parietal cortices orthogonalize the representation of different streams of information concurrently coded in visual working memory
Source: PLoS Biol. 2024 Nov 21;22(11):e3002915. doi: 10.1371/journal.pbio.3002915 (PMC11620661; doi:10.1371/journal.pbio.3002915)
Supplement: S10 Fig — In each ROI plot, the light gray vertical bars mark the stimulus presentation time during the encoding and probe periods, and the medium gray vertical bars mark the fMRI decoding period for the VWM encoding and delay periods. See Materials and methods for more details. The horizontal dashed line indicates chance level decoding. The lighter-colored ribbons around the plot lines represent SE. Data are available at osf.io/8rbkh/. Note for this figure: The single probe object shown at the end of the delay was randomly chosen between the 2 target object types for both change and no change trials (i.e., it was either an exact repeat or another exemplar from the same type of objects). Across trials in the same condition, efforts were not made to show an equal number of each of the 2 target object types as probes. This may explain why during the probe period (the second peak in S10 Fig) cross-decoding may be lower than within-decoding for the target objects; i.e., to decode AC vs. BC, if C does not appear equally often as probes in AC and BC trials, C may contribute to AC vs. BC decoding at the probe stage, resulting in a cross-decoding drop when the AC-BC trained decoder is used to decode AD vs. BD at the probe stage. (PDF) [file pbio.3002915.s010.pdf]

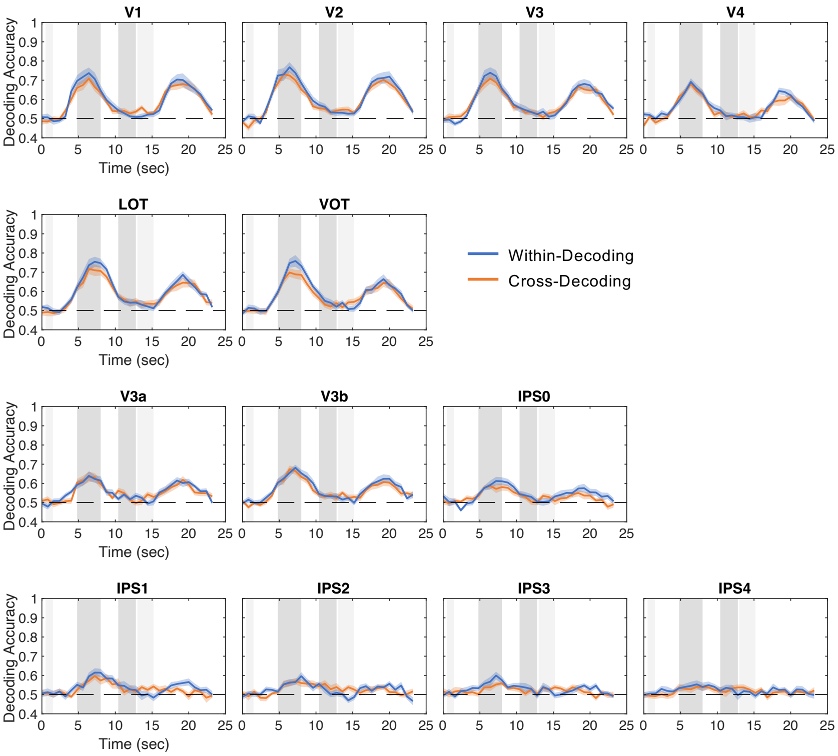

<u>S10 Fig.</u> Experiment 2 target within- and cross-decoding across time. In each ROI plot, the light gray vertical bars mark the stimulus presentation time during the encoding and probe periods, and the medium gray vertical bars mark the fMRI decoding period for the VWM encoding and delay periods. See Methods for more details. The horizontal dashed line indicates chance level decoding. The lighter-colored ribbons around the plot lines represent s.e**.** Data are available at osf.io/8rbkh/.

The single probe object shown at the end of the delay was randomly chosen between the two target object types for both change and no change trials (i.e., it was either an exact repeat or another exemplar from the same type of objects). Across trials in the same condition, efforts were not made to show an equal number of each of the two target object types as probes. This may explain why during the probe period (the 2nd peak in Supplementary Figure 10) cross-decoding may be lower than within-decoding for the target objects. That is, to decode AC vs BC, if C does not appear equally often as probes in AC and BC trials, C may contribute to AC vs BC decoding at the probe stage, resulting in a cross-decoding drop when the AC-BC trained decoder is used to decode AD vs BD at the probe stage.
