# Supplementary material for: The human posterior parietal cortices orthogonalize the representation of different streams of information concurrently coded in visual working memory
Source: PLoS Biol. 2024 Nov 21;22(11):e3002915. doi: 10.1371/journal.pbio.3002915 (PMC11620661; doi:10.1371/journal.pbio.3002915)
Supplement: S11 Fig — (A) Representational space for targets. Each representational space geometry is an MDS projection of the group-averaged RDM of the 4 conditions included. Here, 2 types of targets (pink and purple) are paired with 2 other types of target (black and gray lines). In a given trial, 1 target (pink or purple) is shown with another target (black or gray line) (see Fig 2A). See main text for more details. (B) Target-target angles. In each plot, angles calculated from the RDMs of the individual participants are shown on the left and those from the group RDMs are shown on the right. See main text for more details. Error bars indicate SE. Data are available from S1 Data and at osf.io/8rbkh/. (PDF) [file pbio.3002915.s011.pdf]

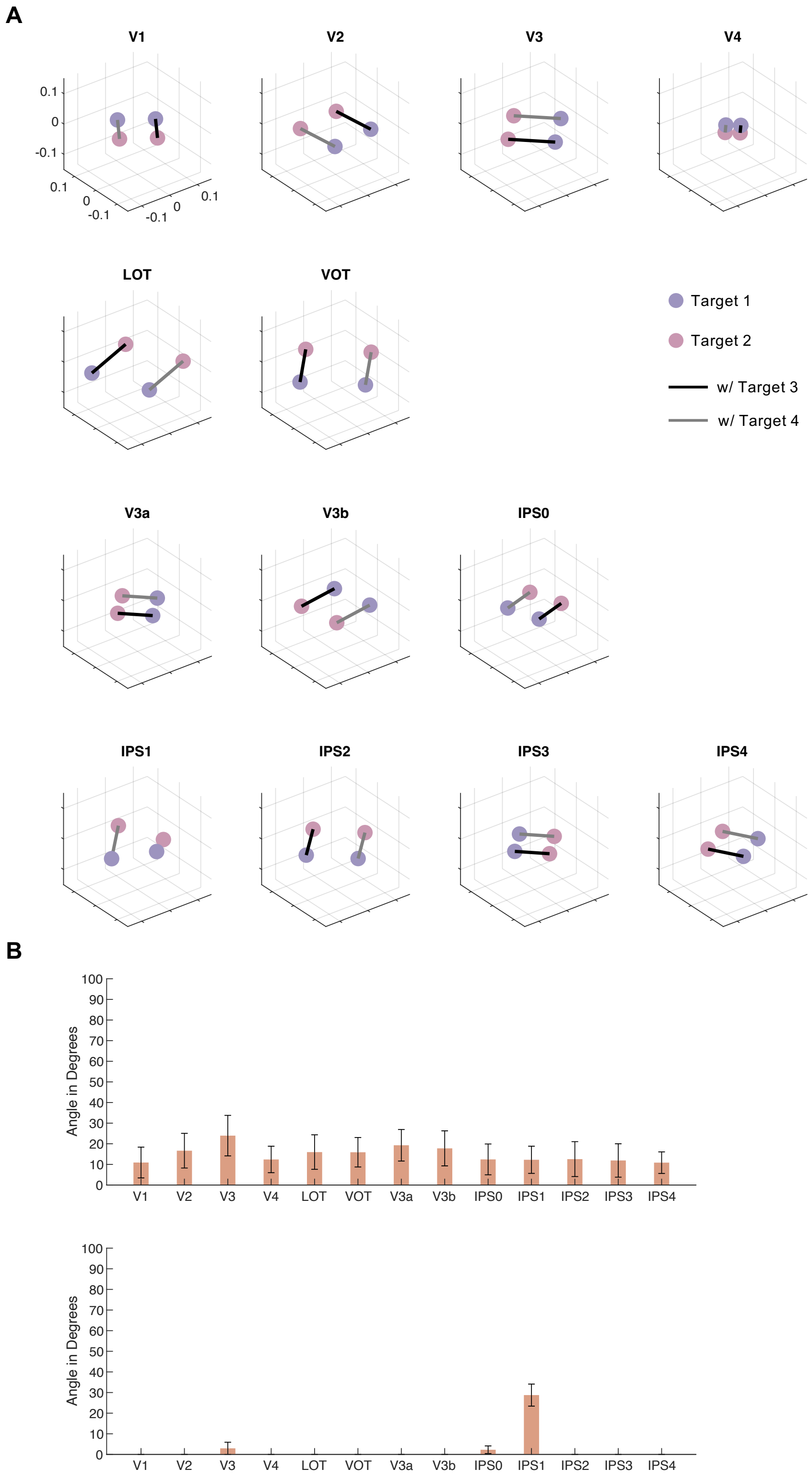

<u>S11 Fig.</u> Experiment 2 angles of target-target representations during VWM delay for each ROI. **A.** Representational space for targets. Each representational space geometry is an MDS projection of the group-averaged RDM of the four conditions included. Here two types of targets (pink and purple) are paired with two other types of target (black and gray lines). In a given trial, one target (pink or purple) is shown with another target (black or gray line) (see Figure 2A). See main text for more details. **B.** Target-target angles. In each plot, angles calculated from the RDMs of the individual participants are shown on the left and those from the group RDMs are shown on the right. See main text for more details. Error bars indicate s.e. Data are available from the supplemental data file and at osf.io/8rbkh/.
