# Supplementary material for: The human posterior parietal cortices orthogonalize the representation of different streams of information concurrently coded in visual working memory
Source: PLoS Biol. 2024 Nov 21;22(11):e3002915. doi: 10.1371/journal.pbio.3002915 (PMC11620661; doi:10.1371/journal.pbio.3002915)
Supplement: S12 Fig — (A) Representational space for targets, as in S11A Fig. (B) Target-target angles, as in S11B Fig. In each plot, angles calculated from the RDMs of the individual participants are shown on the left and those from the group RDMs are shown on the right. See main text for more details. Error bars indicate SE. Data are available from S1 Data and at osf.io/8rbkh/. (PDF) [file pbio.3002915.s012.pdf]

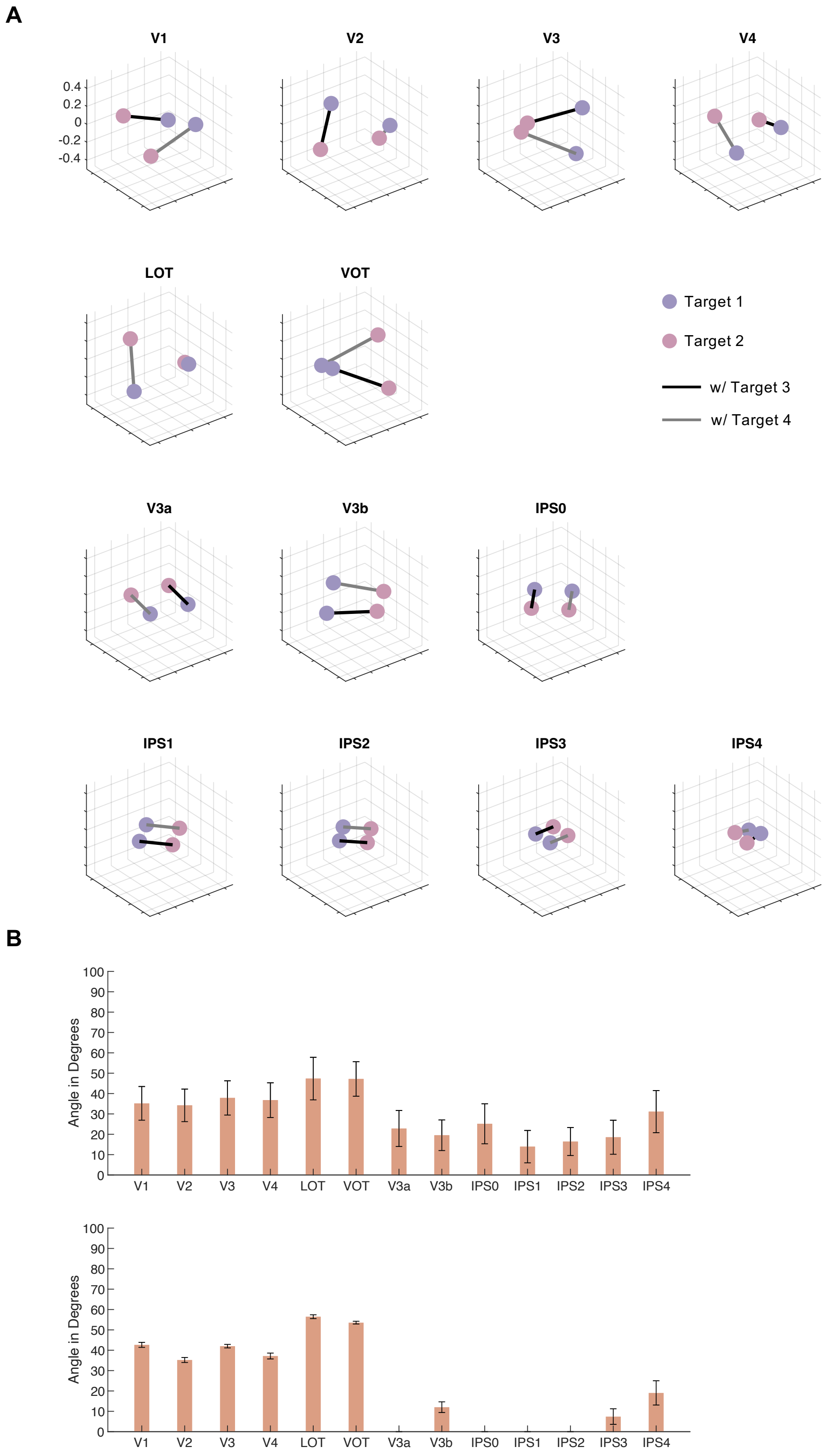

S12 Fig. Experiment 2 angles of target-target representations during VWM encoding for each ROI. **A.** Representational space for targets, as in Figure 11A. **B.** Target-target angles, as in Figure 11B. In each plot, angles calculated from the RDMs of the individual participants are shown on the left and those from the group RDMs are shown on the right. See main text for more details. Error bars indicate s.e. Data are available from the supplemental data file and at osf.io/8rbkh/.
