# Supplementary material for: The human posterior parietal cortices orthogonalize the representation of different streams of information concurrently coded in visual working memory
Source: PLoS Biol. 2024 Nov 21;22(11):e3002915. doi: 10.1371/journal.pbio.3002915 (PMC11620661; doi:10.1371/journal.pbio.3002915)
Supplement: S1 Supplementary Results — (DOCX) [file pbio.3002915.s013.docx]

**Supplementary Results**

Simulation of decoding performance and signal strength

In Experiment 1, decoding performance only needed to be compared during the delay period, and it was fairly well-matched across the different ROI sectors (see Fig 1F and 1I). In Experiment 2, however, decoding performance had to be compared within delay, within encoding, and between encoding and delay. Decoding will inevitably be higher during encoding than delay due to the physical presence of visual stimuli during encoding and be higher during encoding in OTC than PPC due to the sensory nature of OTC. This raises an important question: Would it be valid to directly compare cross-decoding drop given the different levels of within-decoding strength? If the relationship between decoding performance and the underlying pattern signal strength is linear, then the amount of cross-decoding drop directly reflects the amount of signal change, such that adding X amount of signal would increase decoding performance by Y amount regardless of the baseline decoding strength (low or high). Under such a linear relationship, a direct comparison of cross-decoding drop across different levels of within-decoding performance would be valid. However, if the relationship is nonlinear, then such a comparison would not be appropriate. This can happen at very high levels of decoding when decoding performance is saturated and close to one, with an additional increase in signal strength resulting in very little change in decoding performance (e.g., when there is sufficient pattern separation to perfectly differentiate two classes of patterns, additional separation would not further improve the decoding performance).

To directly quantify the relationship between decoding performance and the underlying pattern signal strength, a simulation was carried out. Specifically, two random patterns A and B were first created, each containing 1500 units (chosen to match the lower-end average number of vertices in the brain ROIs, see S2A and S8A Figs). Decoding of A vs B was then performed at different levels of signal strength, from decoding 1% of A and B to decoding 40% of A and B, at a 1% increment (decoding approached 1 at 20% of signal strength). For each decoding analysis, to generate sufficient patterns, 16 samples for A and 16 samples for B were created to mimic the data obtained in a typical fMRI decoding study. At a given signal level of *s*, a pattern for A would be generated by adding *s%* of A and *(1-s)%* of a randomly generated noise pattern, with the noise pattern being different for each of the 16 A and B samples. Decoding was then performed on these 16 A and 16 B patterns. For a given pair of patterns A and B at a given signal level, this simulation was repeated 1,000 times and the results were averaged.

S4 Fig shows 10 simulation cases, each starting with two new randomly initialized A and B patterns. As is evident in the figure, once decoding rises above chance (.5) starting at 5% signal strength (with a decoding accuracy of about .51-.52), a linear relationship holds for the majority of the signal strengths. At the very high signal strengths, there indeed exists a nonlinear relationship and saturated decoding accuracies. Importantly, a probit transformation (i.e., transformation by the inverse of the normal function) is able to remove the nonlinearity and returns a mostly linear function regardless of the signal strength, except when decoding is at chance when the signal strength is less than 5%. If we assume that fMRI noise is largely random, then a probit transformation of decoding accuracy should allow the recovery of a more linear relationship between decoding accuracy and the underlying signal strength, enabling more valid statistical comparisons between conditions when baseline decoding accuracies differ. Thus, in all the decoding results reported in the present study, stats for both the untransformed and the probit-transformed decoding accuracies were included. Results were virtually identical with and without such transformation, if not stronger with the transformed accuracies in some cases.
