# Supplementary material for: The human posterior parietal cortices orthogonalize the representation of different streams of information concurrently coded in visual working memory
Source: PLoS Biol. 2024 Nov 21;22(11):e3002915. doi: 10.1371/journal.pbio.3002915 (PMC11620661; doi:10.1371/journal.pbio.3002915)
Supplement: Attachment — Submitted filename: ReplyLetter_v1.pdf [file pbio.3002915.s015.pdf]

I thank the editor and reviewers for their time and effort, and their very detailed and thoughtful comments on the manuscript. I have performed additional analyses as requested, addressed in detail each comment raised by the reviewers below, and have revised the manuscript accordingly. The major changes made to the manuscript are the following:

- Using multidimensional scaling, I am now able to directly visualize the representational geometry and measure the angles of target-distractor representations in Experiment 1 and target-target representations in Experiment 2. This enables me to directly quantify the orthogonality of the representational space. I am happy to report that these new measurements confirm the results obtained with the cross-decoding analysis. I hope this alleviates the main reservations of both reviewers.

- Reviewer 2 raised the concern that cross-decoding drop may not be detectable when baseline within-decoding is low. I have run a simulation to show that (1) there is a fairly linear relationship between signal strength and decoding performance throughout most of the decoding range, and (2) where nonlinearity is present at the lower and higher ends of decoding, a probit transformation can correct this and that the results are virtually the same, if not stronger, with probit-transformed decoding accuracies.

- I have moved Experiment 2 target presentation order decoding from the supplementary results to the main results, as this result is now extensively discussed in the manuscript per Reviewer 1's comment.

- I have expanded Introduction and Discussion to thoroughly address the other comments raised by the reviewers

- I have removed Figure 1 and Figure 3C as they are not absolutely necessary and have caused unintended confusion for the reviewers.

I hope these revisions have substantially improved the manuscript and have satisfactorily addressed all of the reviewers' concerns. For easy referencing, I include the reviewers' original comments in the italicized text below.

****

Reviewer #1:

This manuscript describes two fMRI experiments in which subjects performed delayed recognition visual WM for real-world objects—bikes, shoes, hangers, couches—with delay-spanning distraction (Exp. 1) or a load of two items, one drawn from each of two categories, and presented serially (both centrally). The motivation is to assess the idea that items processed simultaneously (either in WM with concurrent distraction, Exp. 1; or two in WM, Exp. 2) are encoded

orthogonally. The idea is interesting, although perhaps not as novel as is suggested in the manuscript, but the analytic approach is not clear to this reviewer, and I worry that the interpretations of the results are overstated (best case) or inherently invalid (worst case).

I thank the reviewer for raising these concerns. In the replies below and in the revised manuscript, I have attempted to thoroughly address these concerns.

In my review of the literature (including the references that the reviewer has provided), orthogonal target-distractor and target-target representations in VWM have not been previously established in the human PPC. Among the references that the reviewer kindly provided, a majority of them addressed issues that are not the focus of the present investigation, such as how targets in VWM with different priorities may be coded during VWM delay (Rust & Cohen, 2022; Xie et al., 2022; Piwek et al., 2023; Wan et al., 2022; Want et al., 2024); attention-related, but not VWM-related, processing (Jahn et al., 2024); the rotation between sensory input and LTM-stored information (Libby & Buschman, 2021); and interaction between attention and VWM (Panichello & Buschman, 2021). Degutis et al. (preprint) does address a similar issue to that raised in the present study, but this study has a number of drawbacks as detailed in a later comment below. As discussed in the original manuscript, the results of Ritz and Shenhav (2024), although highly relevant, also suffer a significant confound. As such, it is fair to say that previous research has not rigorously investigated and firmly established the orthogonality of target-distractor and target-target representations in VWM in the human PPC.

The conclusions from these experiments are based on decoding results—can a classifier trained on condition A decode the same stimuli from condition B—and differing levels of cross-classification results are interpreted as evidence for different levels (or, in some cases different kinds) of representational geometry. This approach, although not inherently flawed, is necessarily indirect, in that representational geometry is never directly measured. Thus, a statement such as the first sentence of the Discussion—" Using fMRI pattern decoding, this study unveils a common representational geometry that our brain uses to combat distractions in VWM"—can't literally be true, because "to unveil" implies to describe (and ideally to quantify) the geometry itself. The best that decoding can do is support inferences about "what the geometry might be." Admittedly this level of description and quantification is easier with invasive physiological measures (e.g., [1-5]) or with artificial neural networks (e.g., [6, 7]), but it can be done (e.g., [8]), and it doesn't help the credibility of analyses with human neuroimaging data if the claims are exaggerated.

I agree with the reviewer that direct quantification of the representational geometry is easier with neurophysiology and artificial neural networks than with neuroimaging. Nevertheless, although cross-decoding can only infer the representational geometry, it can be used to test key properties and predictions of the representational geometry and allow us to form a valid description of the underlying geometry. For example, the large

body of work by DiCarlo and colleagues is based on cross-decoding results and has significantly advanced our understanding of the object representational geometry in the primate ventral visual cortex (DiCarlo et al., 2006; DiCarlo et al., 2012).

That being said, I took the reviewer's comments to heart and have been able to directly visualize the representational geometry using multidimensional scaling (MDS). Specifically, because each cross-decoding analysis involves 4 conditions (e.g., training on A and B to cross-decode C and D and vice versa), instead of performing cross-decoding, we can extract pairwise similarity measures across the four conditions and use MDS to project the resulting representational dissimilarity matrix into a 3D space. Since only four conditions are included, a 3D space can fully capture all the representational variance in the similarity structure with no variance left unexplained. Here in the 3D space, we can directly measure the angles of the representational geometry and quantify the orthogonality of representation. These new results are reported in the revised manuscript and, critically, they confirm the cross-decoding results (see pp.11-13 and pp.18-20 of the revised manuscript, Figures 2 and 4, and Supplementary Figures 6, 7, 11 and 12).

> *A more substantive concern for this reviewer is that I simply can't understand the inferential logic of the approach. For Experiment 1: What's being decoded is bike from hanger. If train bike/hanger [couch distractor] --> test bike/hanger [shoe distractor] is lower, this suggests that bike & hanger are represented differently with bike distractor than with shoe distractor. It doesn't speak to whether, say, bike and couch are orthogonalized. To be explicit, let's assume that the truth is that memory item and the distractor are held in orthogonal subspaces, in this scenario it could be the case that the representational geometry of bike is the same regardless or distractor (in which case cross-decoding performance would be quite high), or it could be the case that its representational geometry is different when in a 'couch' context than when in a 'shoe' context (in which case cross-decoding performance would be low). Thus, levels of cross-decoding performance is not diagnostic of relation between memory item and distractor. The same reasoning holds for Experiment 2. It may be that I'm missing something that would argue against this reasoning, but if so I can't find it anywhere in the manuscript. One does get the sense while reading the manuscript that the author is relying heavily on methods used and conclusions drawn from her previous work, but this manuscript lacks the specific information to preempt the reader from drawing the conclusion that I have drawn here. (One also gets the sense that Figure 1 is supposed to explain the logic behind the analyses, but I don't understand it. What are the digits? Where is there an illustration of orthogonal representations? Where is the "representational geometry"?)*

As the reviewer stated, if the VWM target and distractor are held in orthogonal subspaces, then cross-decoding performance would be quite high; but when they are not, then cross-decoding performance would be low. Thus, by assessing cross-decoding performance, we can infer the structure of the target and distractor

representational space. With MDS, we can now directly visualize the representational geometry to confirm the orthogonality in representation. The reviewer's statements and reasoning thus appear to agree with the conclusions of the study, rather than contradict them. So, I am not entirely understanding the reviewer's concern here.

One possible alternative interpretation of the reviewer's comment is that even though target and distractor representations can remain orthogonal and result in no cross-decoding drop, this may be accomplished in two ways: (1) target representations remain fixed and are sustained by a subset of neurons; or (2) targets and distractors are coded by the same neurons, and although target representations can vary in different distractor contexts, target representations remain orthogonal to those of the distraction condition in the representational space formed by the neuronal population, thereby still enabling target information to be read out independently of distraction by downstream regions. With fMRI pattern responses summing over the neuronal population, both (1) and (2) would produce the same results. Further research involving direct neuronal recording is thus needed to distinguish between these two representational possibilities. This discussion has been added to the revised manuscript on pp 22-23.

It is worth noting that these two ways of representation echo our evolving understanding of how object identity information is coded in primate high-level vision. To explain how we can recognize objects despite changes in viewing conditions involving features such as position, size and viewpoint, it was initially thought that the primate IT cortex (the apex of ventral visual processing) must contain view-invariant object identity neurons. However, neuronal recording studies show that IT neurons in fact encode both object identity and non-identity features (such as position, size and viewpoint), as these non-identity features, although nonessential in object recognition, are part of the visual input and are important in our interactions with the objects. Critically, DiCarlo and colleagues found that information in the IT cortex is organized in such a way that would still allow the readout of the object identity information (see DiCarlo & Cox, 2007; DiCarlo et al., 2012 for a review of this literature). Specifically, instead of having grandmother cell-like identity neurons, in the representational space formed at the neuronal population level, the IT cortex represents both object identity and non-identity information in a near orthogonal manner (cross-decoding is always a little less than within-decoding, see Xu & Vaziri-Pashkam, 2022), allowing downstream regions access to object identity information largely independent of non-identity information. Based on this line of research, I suspect that option (2), rather than (1), may also apply to how targets and distractors are coded in PPC, although direct neuronal recording data are needed to test this.

In Figure 1, each number indicates a target and a distractor pairing. Given the unintended confusion this figure has caused, it has been removed from the revised manuscript.

> *This is a fast-moving field, and it's understandable that not every scientist will be familiar with every new finding, but there are some missing from here that might help fill some holes. For example, the statement that "it is unclear how targets*

> *may be differentiated when location coding is not available, such as when targets are show sequentially at the same location" is contradicted by at least two studies [5, 9], both of which describe a scheme that is effectively what the author is proposing here, albeit with much more detail and quantification. For example, both illustrate that 'earlier' rank subspaces account for less variance than those representing the more recently presented item, thereby also preceding this manuscript in suggesting that reduced information with increasing rank may account for visual WM capacity limitations. The author will also be interested to see this preprint, which addresses many of the same questions [8].*

I thank the reviewer for mentioning these studies which I am aware of. [5] requires the encoding of item sequence in WM. [9] assigns priority to the two items encoded in WM, again making the encoding order task-relevant. Thus, in both studies, the encoding order of the items needs to be explicitly remembered, making the coding schemes reported in both studies likely task-induced. In contrast, in Experiment 2 of the present study, the encoding order was never required or task-relevant. Thus, [5] and [9] do not speak directly to the present study and it is very possible that order may not be represented when it is task-irrelevant, especially in a task-driven region like PPC. It is from this perspective that I made the above statement. I have added this discussion on the revised manuscript on p.24. I thank the reviewer for raising this point.

I am aware of [8], but since it was posted on May 23, more than one month after the current manuscript was submitted, I was not able to discuss it in the submitted manuscript. While [8] asks some of the same questions, there are a few drawbacks. For example, [8] did not have extensive coverage of PPC ROI (only to IPS1), and thus did not cover higher PPC areas. While [8] found an about 7-degree angle difference between the target and distractor subspaces in a macro V1-V3ab ROI, this is far from being orthogonal. In the present study, I am able to extract the angle in each ROI and found small angles in early visual areas but a close to 90-degree angle in higher PPC areas (derived from parallel target-target and distractor-distractor representations; see Figure 2 of the revised manuscript), showing true orthogonality in those areas. [8] found no cross-decoding drop in some of the early visual areas. However, this would suggest a close to 90-degree angle difference between the target and distractor subspaces, contradicting the 7 degrees of angle mentioned above. Because only six subjects were included (in contrast to 14 in Experiment 1 of the present study), [8] may not have the power to detect small but significant effects. [8] used non-parametric permutation tests to test the significance of the cross-decoding drop, but details of these permutation tests were not given. If results from all six subjects were averaged first before the tests were conducted in a fixed-effects analysis (rather than the mixed-effects analysis as used in the present study), then some of the significant results may be driven by a small subset of the subjects. This again puts less confidence in some of the conclusions reached. That being said, the study is relevant and I have included a brief discussion of this study in the revised manuscript on pp.21-22.

> *Finally, the word "untangling" is used a lot, but for it to be useful it needs to be defined as a precise operation and/or state. What does it mean for information to*

get "tangled"? I would be concerned about confusion with the concept of entanglement from nonlinear dynamical systems, which has a very different meaning than what I think is intended here, and is also making its way into systems and cognitive neuroscience (e.g., [10, 11]).

The reviewer has a valid concern here and I have made more explicit what I meant by untangling. Specifically, I borrow the term from the object processing literature, in which untangled representations would allow independent read-out of the different representations, whereas tangled representations would not. I have added this in the revised manuscript on p.4.

1. Jahn, C.I., et al., Learning attentional templates for value-based decision-making. Cell, 2024.
2. Libby, A. and T.J. Buschman, Rotational dynamics reduce interference between sensory and memory representations. Nature Neuroscience, 2021.
3. Panichello, M.F. and T.J. Buschman, Shared mechanisms underlie the control of working memory and attention. Nature, 2021. 592: p. 601-605.
4. Rust, N.C. and M.R. Cohen, Priority coding in the visual system. Nature Reviews Neuroscience, 2022. 23: p. 377-388.
5. Xie, Y., et al., Geometry of sequence working memory in macaque prefrontal cortex. Science, 2022. 375(6581): p. 632-639.
6. Piwek, E.P., M.G. Stokes, and C. Summerfield, A recurrent neural network model of prefrontal brain activity during a working memory task. PLoS Comput Biol, 2023. 19(10): p. e1011555.
7. Wan, Q., J.A. Menendez, and B.R. Postle, Priority-based transformations of stimulus representation in visual working memory. PLoS Computational Biology, 2022. 18(6): p. e1009062.
8. Degutis, J.K., et al., Neural dynamics of visual working memory representation during sensory distraction. preprint.
9. Wan, Q., et al., Representing context and priority in working memory. Journal of Cognitive Neuroscience, 2024. in press: p. tbd.
10. Pessoa, L., The Entangled Brain. J Cogn Neurosci, 2023. 35(3): p. 349-360.
11. Pessoa, L., Disentangling some conceptual knots. Journal of Cognitive Neuroscience, 2023. 35: p. 391-395.

I thank the reviewer for these references and have added a subset of these that are most relevant to the present study to the revised manuscript.

Reviewer #2:

The study investigates brain mechanisms resolving interference during visual working memory, specifically, whether early (EVC), occipitotemporal (OTC) and parietal (PPC) cortices encode and maintain target and distractor (or target and target) representations in linearly independent subspaces. In Experiment 1,

participants perform a 1-item memory task, but the delay period is either filled with a series of distractor objects or remains blank. To test for an independence of target and distractor representations in each area, Xu trains a classifier to differentiate memorized targets on trials with one distractor and tests it on trials with another distractor (or a blank delay). The cross- vs. within- condition comparisons indicate worse target-distractor separability in EVC and OTC compared to PPC. In the Experiment 2, participants memorize two concurrently presented items (without distraction). Using similar cross-decoding strategy, Xu shows that during encoding, information about targets is less separated in EVC and OTC than in PPC, similar to Experiment 1. During maintenance, this difference is not observed any more, indicating a contribution of a feedback from PPC to OTC and EVC during the memory delay.
I found this manuscript an interesting read, and the topic is very timely. However, the text is hard to discern and there are many typing errors. The sentences need to be simplified where possible and the grammar must be checked throughout.

I thank the reviewer for the positive comments made here. I apologize for the typo and grammar errors. The revised manuscript has been carefully read by a native English speaker and hopefully all errors have been corrected.

Major:
1). If linear decoder generalizes across conditions, this does not mean that the subspaces it separates are orthogonal. Successful cross-decoding indicates that the memory subspace is consistent across distractors, however the orthogonality claim requires measuring of the angles between the target and distractor subspaces, and one cannot infer such relationship from cross-decoding only. Therefore, I suggest that the author adds orthogonality tests or, otherwise, changes the inferences accordingly.

Using MDS, I am now able to directly visualize the representational geometry and measure the angles of representation to confirm orthogonality of target and distractor representations (see Figures 2 and 4, Supplementary Figures 6, 7, 11 and 12, and the revised manuscript on pp.11-13 and pp.18-20). I thank the reviewer for raising this point.

2). I find the sample sizes very small in both experiments, therefore, I'd like to ask for the power analysis for the assumed effects. For example, in the target-target cross decoding analysis (Experiment 2), the difference between the within and cross-decoding in the delay phase of the trial is much weaker overall than the results from the encoding phase and this might explain the absence of the difference during the delay (due to lack of statistical power and not feedback from PPC as the current interpterion holds). Same concern applies for the EVC/OTC vs IPS comparisons: the overall decoding in IPS is much weaker than in the other areas. The difference between the within- and cross-decoding seems to be present, but it might not reach significance due to the underpowered design.

Despite the reviewer's concern, the sample sizes of both experiments are actually higher than or similar to other recently published studies in high-profile journals (e.g., Ns = 6 and 7 for the two experiments in Rademaker et al., 2019, *Nature Neuroscience*; N = 13, in Yu … Postle, 2020, *PLOS Biolog*y; N = 16 in Yu … Buschman, 2020, *PLOS Biolog*y; N = 7 in Hallenbeck et al., 2021, *Nature Communications*; Ns = 13 and 14 for the two experiments in Li et al., 2021, *Neuron*; N = 11 for Kwak & Curtis, 2022, *Neuron*; N = 14, Li & Curtis, 2023, *Current Biology*).

It is worth noting that during VWM delay the overall within-decoding level was similar between Experiments 1 and 2 and, importantly, across both ventral and dorsal regions. Yet while cross-decoding was capable of dropping in Experiment 1 in ventral regions, it did not drop in Experiment 2. In Experiment 2 during the delay period, the average cross-decoding was numerically higher than within-decoding in both V1-V4 and IPS2-4; although cross-decoding was numerically lower than within-decoding in LOT/VOT, power analysis (at a power of .80) shows that to make this difference significant (one-tailed), we will need 449 subjects. Meanwhile, both within- and cross-decoding are significantly above chance in all three ROI sectors. Thus, the lack of a cross-decoding drop in all three ROI sectors does not appear to be due to a lack of power, but rather it reflects a similar level of within- and cross-decoding performance. I have added this discussion to the revised manuscript on pp.16-17.

Decoding will inevitably be higher during encoding than delay due to the physical presence of visual stimuli during encoding and higher during encoding in ventral than dorsal regions due to the sensory nature of the ventral regions. Thus, the question at heart is, would it be valid to directly compare cross-decoding drop given the different levels of within-decoding strength? If the relationship between decoding performance and the underlying pattern signal strength is linear, then the amount of cross-decoding drop would directly reflect the amount of signal change, such that adding X amount of signal would increase decoding performance by Y amount regardless of the baseline decoding strength (low or high). Under such a linear relationship, a direct comparison of cross-decoding drop across different levels of within-decoding performance would be valid. On the other hand, if the relationship is nonlinear, then such a comparison would not be appropriate. This can happen at very high levels of decoding when decoding performance is saturated and close to one, with additional increase in signal strength resulting in very little change in decoding performance (such as when there is sufficient separation to perfectly distinguish two classes of patterns; additional separation would not improve decoding performance). The question remains: Does a similar nonlinear relationship hold at very low levels of decoding, with increasing signal strength failing to proportionally increase decoding performance? I believe it is this potential nonlinear relationship that the reviewer is concerned about.

Because I am unaware of any study in the brain imaging community that has directly quantified the relationship between decoding performance and the underlying pattern signal strength, I decided to run a simulation. Specifically, I first created two random patterns A and B, each containing 1500 units (chosen to match the lower-end average number of vertices in the brain ROIs, see Supplementary Figures 2A and 8A). I then

performed decoding of A vs B at different levels of signal strength, from decoding 1% of A and B to decoding 40% of A and B, at 1% increment (decoding approached 1 at 20% of signal strength). For each decoding analysis, to generate sufficient patterns, I created 16 samples for A and 16 samples for B to mimic the data obtained in a typical fMRI decoding study. At a given signal level of s, a pattern for A would be generated by adding s% of A and (1-s)% of a randomly generated noise pattern, with the noise pattern being different for each of the 16 A and B samples. Decoding was then performed on these 16 A and 16 B patterns. For a given pair of patterns A and B at a given signal level, this simulation was repeated 1,000 times and the results were averaged. The attached figure shows 10 simulation cases, each starting with two new randomly initialized A and B patterns.

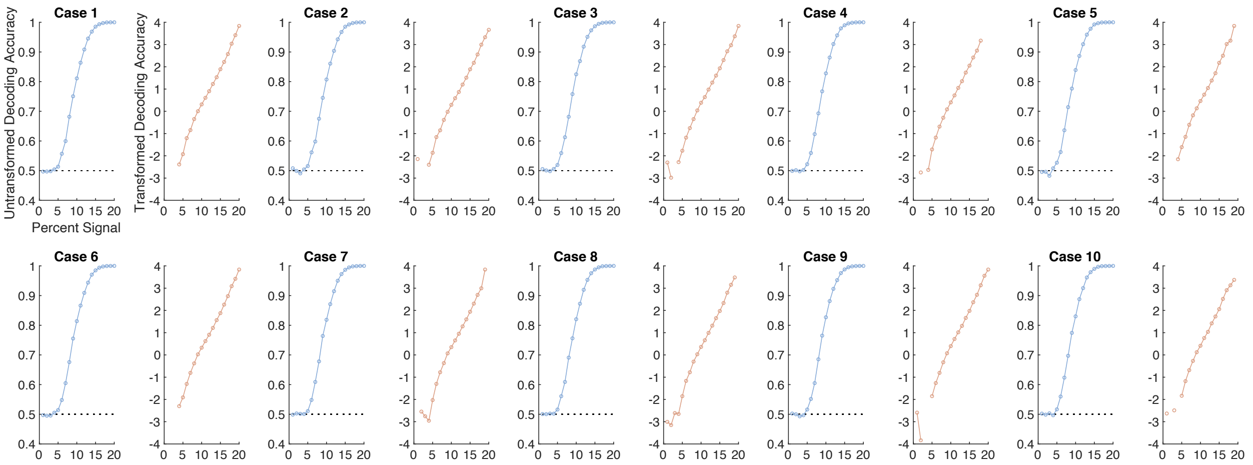

As shown in the figure (blue lines), once decoding rises above chance (.5) starting at 5% signal strength (with a decoding accuracy of about .51-.52), a linear relationship holds for the majority part of the signal strengths. At the very high signal strengths there indeed exists a nonlinear relationship and saturated decoding accuracy. Importantly, a probit transformation (i.e., transformation by the inverse of the normal function) is able to remove the nonlinearity and returns a mostly linear function regardless of the signal strength, except when decoding is at chance when the signal strength is less than 5%. If we assume that fMRI noise is largely random, then a probit transformation of decoding accuracy should allow us to recover a more linear relationship between decoding accuracy and the underlying signal strength, enabling more valid statistical comparisons between conditions when baseline decoding differs. Thus, in all the decoding results reported in the revised manuscript, I now included stats on both the untransformed and the probit-transformed decoding accuracies. Results were virtually identical with and without such transformation, if not stronger with the transformed accuracies in some cases. The simulation procedure is described in Supplementary Results and the simulation results are reported in Supplementary Figures 4.

I hope the simulation results and probit-transformed decoding results address the reviewer's concern. I thank the reviewer for bringing up this comment. This is one of the assumptions of MVPA that has not been thoroughly tested to my knowledge. It is

necessary and important to document this. I thank the reviewer for bringing up this important point.

3). It would be helpful if the author could relate the current findings to the size of the receptive fields in the compared areas, in particular, how representational overlap may contribute to the stability and separability of the memorized items.

This is an interesting idea to consider. Higher visual areas and PPC are known to have larger receptive field sizes than EVC (Klein, Harvey, & Dumoulin, 2014, *Neuron*). Although receptive field size in LOT/VOT is about 2 or 3 times larger than those in V1 to V3, a similar cross-decoding drop between distraction conditions is observed. Hence, at least in the ventral cortex, increased receptive field size and thus increased representational overlap do not seem to contribute to the stability and the separability of the memorized items. Receptive field size is more or less constant across PPC areas, and yet we are seeing stability and separation in higher rather than lower PPC areas, again showing that the size of the reception field may not matter there. If anything, I would think conceptually that smaller receptive field size and thus less overlap in representation may better separate the representations. But this does not appear to be the case. So, I am unsure that the size of the receptive field can account for the present results. But perhaps I am missing something here and it would be great if the reviewer could elaborate on this point more.

Minor:
1). All the within ROI results (V1, V2, V3 and V4 in EVC) must be at least corrected for the number of small ROIs included.

Not sure I exactly follow this suggestion. How may the correction be done? The results are averaged over the individual ROIs for each participant before stats are performed at the group level. Within- and cross-decoding results for V1-V4 are all *ps* < .01. If we apply the Benjamini-Hochberg method and multiply the p-values by the number of areas included in EVC, we will still have at least *ps* < .05 for all the comparisons. This would not change any of the conclusions reached.

2). I did not understand the purpose and explanation of the figure 3C.

This was a figure borrowed from DiCarlo et al. (2012) which was used to explain representational straightening in the IT cortex for representing object identity and non-identity information. Using the same logic, here I was arguing that in OTC, target and distractor representations are entangled, preventing a linear decoder from reading out the target information independent of the distractor condition. In PPC, however, with the straightening of the target and distractor representational space, a linear decoder can have access to the target information independent of distraction. To avoid confusion, I have removed this figure from the revised manuscript as it is not essential in helping explain the results.

3). I did not understand the assumed role of synchronization in resolving representational interference in the Discussion.

The idea of synchronization is that features of the same object are synchronized in phase together, with different phases of the synchronization containing features from different objects. There is thus a limit to how many total objects may be represented simultaneously. This has been argued to be the source of VWM capacity limitation. I have clarified this on p.24 of the revised manuscript.

4). In Experiment 2, the change in the decoding drop at encoding phase to the no decoding drop at maintenance phase needs to be statistically compared to interpret the absence of the difference at the latter phase.

This is a valid point and this comparison has been added in the revised manuscript on pp.17-18.
