# Supplementary material for: The human posterior parietal cortices orthogonalize the representation of different streams of information concurrently coded in visual working memory
Source: PLoS Biol. 2024 Nov 21;22(11):e3002915. doi: 10.1371/journal.pbio.3002915 (PMC11620661; doi:10.1371/journal.pbio.3002915)
Supplement: Attachment — Submitted filename: ReplyLetter_v1.pdf [file pbio.3002915.s017.pdf]

I thank the reviewers for their time and effort, and their very detailed and thoughtful comments during the 2nd round of the review. Below I have addressed each comment in detail and have revised the manuscript accordingly. The major changes made to the manuscript have been marked with track changes. I have also made additional minor stylistic changes to the manuscript to further improve its readability. I hope these revisions have improved the manuscript and satisfactorily addressed all of the reviewers' remaining concerns. For easy referencing, I include the reviewers' original comments in the italicized text below.

****

Reviewer #1:

Reviewer 1:
"Untangling representations in visual working memory in human occipitotemporal and posterior parietal cortices," Xu. This is a revision, and overall I find it to have been very responsive (and the point-by-point cover letter to be very clear and thorough.) In particular, removing the original Figure 1 removed some confusion, and adding the MDS visualizations and the calculation of angles separating representational subspaces now bolsters the interpretation of the decoding results, which support only indirect inference about representational geometry.

The author writes:
"In my review of the literature (including the references that the reviewer has provided), orthogonal target-distractor and target-target representations in VWM have not been previously established in the human PPC. Among the references that the reviewer kindly provided, a majority of them addressed issues that are not the focus of the present investigation, such as ... As such, it is fair to say that previous research has not rigorously investigated and firmly established the orthogonality of target-distractor and target-target representations in VWM in the human PPC."

I don't disagree with this concluding statement, but would also suggest that this is a rather narrow (and very specific) focus. If the author's goal with this paper is to communicate her findings with other scientists studying related questions, an effective way to broaden the potential impact of this work might be to make reference to other work in which a similar scheme (orthogonal representation) has been proposed for somewhat different contexts. E.g., how targets in VWM with different priorities may be coded during VWM delay; attention-related, but not VWM-related, processing ; the rotation between sensory input and LTM-stored information (Libby & Buschman, 2021); and interaction between attention and VWM (Panichello & Buschman, 2021) are all examples of visual cognition, and although what this author is reporting here is strictly limited to "orthogonality of target-distractor and target-target representations in VWM in the human PPC," what she reports will also be of interest to people studying these other kinds of visual cognition.

I agree with the reviewer that representation orthogonalization has been reported in other contexts in visual cognition and it would be useful to include these studies in a broader discussion. I have now included such a discussion in the revised manuscript on p.26. I thank the reviewer for this suggestion.

> *Below I'll add some minor points that the author may want to consider that could help with clarity.*

> *p. 4. "entanglement" Please consider being more explicit about the context in which it is being used, e.g., actually include something along the lines of "I borrowed the term from the object processing literature, in which untangled representations would allow independent read-out of the different representations, whereas tangled representations would not." And then also include citations that support this.*

This is a good suggestion, and I have added the suggested text to the revised manuscript on p.4.

> *Fig. 1.J. These values are not from a time series, but are discrete values, one from each ROI, and so should be plotted discretely (e.g., with same format as the other bar graphs in this figure).*

As the reviewer suggested, the line graph has been changed to a bar graph.

> *Legend to Figure 1. Here, and elsewhere, is it more precise to use "… trained to classify bikes relative to hangers" or "… trained to discriminate bikes from hangers"?*

These texts have been revised following the reviewer’s suggestion.

> *Legend to Figure 1. Panels J and K are not included in the legend.*

My apologies for this omission. The legends for Figure 1 panels J and K have now been added.

> *p. 8. Consider specifying that "Significant VWM decoding OF THE TARGET was obtained …", so as to clarify that this is item decoding, rather than decoding of pairings of stimuli.*

This was indeed the intended meaning. I thank the reviewer for pointing this out. The text has been revised.

> *p. 13: "Thus, only in IPS2-IPS4 we have near 0 … Consider rephrasing to be less colloquial?*

This has been rephrased as “Only IPS2-IPS4 showed a near zero-degree angle …”.

> *p. 13: MDS results don't confirm the cross-decoding results so much as they support your interpretation of those results.*

The sentence in question has been revised to include the more precise wording suggested by the reviewer.

> *Figure 4 and related text: it would be more straightforward to present the encoding results first, then the delay results.*

Because the present study centered on VWM delay period target representations, the delay results were the main focus and thus were presented first. The encoding results were included to provide additional details for the delay results.

> *"Participants" section: the "Yu … X (20xx)" citations should be formatted with "et al." like the others?*

There were two Yu et al. (2000) studies with different lists of coauthors. Yu … Postle, 2020 and Yu … Buschman, 2020 were thus used to discriminate between these two studies. I am happy to let the copy editor decide how to best cite these two studies.

> *Supplementary Figure 3: I found legend difficult to follow, particularly because the 'encoding' bar is difficult to see. Consider including an empty plot that uses written labels and arrows to show what shading and "distraction timeline" correspond to (i.e., almost like a legend for the graphical conventions)?*

This is a great suggestion. I have included this in the revised Supplementary Figure 3.

I thank the reviewer for all the excellent suggestions made above, the implementations of which have further enhanced the manuscript.

> ***Reviewer #2:***

> *This is an interesting topic, the data are high quality, and the experiment was well-executed with multiple sessions collected from n=14. That said, I found the logic of the analyses and the interpretation of the data to be a bit challenging to follow.*

> *I think the author’s interpretation of Experiment 1 boils down to this - (1) in OTC representations of specific target categories change as a function of the distractor category they are paired with, therefore target-distractor representations are entangled, and (2) in IPS2-4 target representations do not change as a function of the distractor they are paired with, therefore target-distractor representations are disentangled.*

If this is the intended interpretation, I would find the paper much easier to digest if simpler terminology were adopted. For instance, nowhere do you need to say "orthogonal", and you could probably get rid of "entangled" and "disentangled" and just say "interacting" and "independent" or something of that sort. This seems like a minor suggestion, but I think it is quite important, especially for a general readership journal and people outside the field are going to struggle. I am quite familiar with this general field, and the work of DiCarlo and Rust and others, and I found that there wasn't a clear statement about the rationale/predictions that link all these terms together in a meaningful way. So, my suggestion is to simplify all the jargony descriptions and to have a very clear statement that spells out the logic of the analysis step-by-step before diving into the results.

I thank the reviewer for this comment. A lot of these terms are included to make contact with the existing literature and are useful to provide a more detailed characterization of the representational space. For example, "entangled" and "disentangled" are terms used by DiCarlo and colleagues to describe object representations in the IT cortex. In my view, these terms provide a more precise description of the representational space than more generic terms such as "interacting" and "independent". Likewise, the term "orthogonal" depicts a very specific representational geometry, useful for providing a precise characterization of the representational space. Since it is possible to provide a quantitative characterization of the representational space with the current data, I believe the usage of more precise terms is helpful, especially for researchers in this field. Nevertheless, I agree with the reviewer that there were a lot of terms, and it could be challenging for readers who are not familiar with this field of research. To remedy this, I have added a paragraph at the end of the introduction on p.6 of the revised manuscript to provide a simplified step-by-step description of the logic of the analysis. I hope this clarifies things and makes the manuscript easier to read for non-expert readers.

The "orthogonal" target-distractor representations in PPC are described as if the orthogonalization occurs via some active and adaptive process. I think an alternative explanation is that PPC just doesn't encode much information about the distractors and therefore the representations don't interact. Supplemental Figure 3C seems to support this, as in higher areas of IPS distractor decoding is about on par with target decoding. In contrast, distractor decoding in all earlier areas is higher than target decoding. What supports the "adaptive orthogonalization" account over an account that posits filtering of distractor information (the alternative account you set up in the intro)? I understand that simulations were performed to address the SNR issue, but here I'm focused not on overall SNR but on the relative SNR of target and distractor representations. Distractors dominate in OTC but are on par with targets in PPC. Thus, it seems like supporting the "adaptive orthogonalization" account over the "distractors are filtered out before PPC" account would require some evidence that the target-distractor representations start entangled and then actively orthogonalize.

Alternatively, you could show that the independence holds across different levels of distractor decoding accuracy. Otherwise, I'm not sure how to tease apart these two accounts?

It is worth noting that although distractor decoding in PPC was on par with target decoding during WM encoding, it was still much higher than target decoding during WM delay. Thus, it may not be accurate to say that PPC does not encode much distractor information. Rather, distractor representation was still much stronger than target representation during delay. The reviewer was correct in pointing out that distractor representation was much greater in OTC than in PPC. Thus, a filtering mechanism could have partially contributed to distractor suppression in PPC. This would echo prior work (including our own) showing that visual representations in PPC are more task-driven than those in OTC (Bracci et al., 2017, *Cereb Cortex*; Vaziri-Pashkam et al., 2017, *J Neuro*). Even so, task-irrelevant sensory information could still be represented robustly in PPC in Vaziri-Pashkam et al. (2017) and the present study. This shows that a filtering mechanism was unable to completely suppress or remove distractor representation. In a VWM setting, the question then comes down to: how would PPC accommodate the representations of targets and distractors, with the latter being much stronger than the former? The present results showed that PPC used an orthogonal representational scheme to accomplish this, which was absent in OTC. Such a representational scheme is adaptative as it enables PPC to maintain the invariance of its VWM representations to distraction while at the same time accommodating distractor representations. The latter can be highly advantageous in situations where distracting information may become task-relevant (e.g., avoiding an obstacle in the road) and in dual-task settings where distractor processing is also task-relevant. PPC may thus use both a filtering mechanism and representation orthogonalization to combat distraction in VWM.

I thank the reviewer for bringing up this discussion point and have added this discussion to General Discussion in the revised manuscript on p.22.

The presence of distractors has no impact on target decoding accuracy. So whatever interactions or entanglement exists between targets and distractor categories doesn't degrade the amount of information about the targets. This seems consistent with a simpler interpretation that early visual areas care more about low-level features and spatial configurations of stimuli, whereas PPC is less selective for these attributes? Again, maybe this is the point the author is trying to make – that distractors have been filtered out before PPC and so PPC is more invariant or stable. However, I am again a bit confused about why the conclusion isn't just that early visual areas are more sensitive to low-level sensory features whereas PPC is more sensitive to relevant features that are attended or stored in memory (consistent with many prior studies). However, in both OTC and PPC, distractors don't lead to a loss of information about the remembered target.

In a recently published study (Xu, 2023, *Curr Bio*), with a set of real-world objects, I showed that during the delay period, the VWM representational content becomes aligned between OTC and PPC, with OTC representation being more similar to that of PPC during encoding and delay than to its representation during encoding. This shows that feedback from PPC can actively shape the representational content of OTC during VWM delay. Since not all features of a complex real-world object are relevant in a VWM task, PPC likely encodes only the task-relevant object features, which are then retained in VWM in PPC and then in OTC through feedback. Given that VWM retention in OTC requires top-down control, with the limited VWM capacity, it is unclear why OTC should always retain low-level features and spatial configurations of stimuli that may not be task-relevant as the reviewer stated above. Results from Clay Curtis' lab further demonstrate that representations in early visual areas can transform from the low-level sensory stimuli initially obtained during VWM encoding to more abstract representations during VWM delay (Kwak & Curtis, 2022, *Neuron*). This is again inconsistent with the view that early visual areas only care more about low-level features.

Decoding accuracy is a very coarse measure of representation: It tells you that the representations of the two targets are still equally distinctive, but it doesn't tell you whether or not the representations are the same with and without distraction. If VWM is to play an important role in guiding our behavior and thoughts, information stored in VWM should be faithfully maintained and should not be easily distorted by distraction (which is ubiquitous in real-world vision). The present study shows that while PPC VWM representations possess such invariance, those in OTC do not. The present results thus have significant implications regarding the differential roles OTC and PPC may play in VWM.

Three more comments just to consider.

1) I think you added this text in response to a prior reviewer comment, but I would not suggest including your alternative account of another's work based on speculation about the methods that they used in a non-peer-reviewed version of their paper (Degutis). This seems unnecessary as their paper is not published could prove awkward down the road if your presumption is incorrect. You could email them to ask, or to see if they have an updated version – especially a version of record – but I would personally endorse removing that passage.

Degutis et al. has not been published and there was no update on that manuscript. I agree with the reviewer's point here and have removed that paragraph. It was included during the last round of revision as one of the reviewers asked for an explicit discussion of that study.

2) This may reflect something that the other reviewers know and I am missing, but I don't think the angle measurements add much beyond the cross-decoding analysis. If there is a drop in cross-decoding, there is a rotation, if not, then no rotation. Perhaps removing some of the "orthogonal" jargon would make these analyses seem even less necessary. Again, apologies if I'm missing something.

During the previous round of review, the reviewers brought up the concern that a cross-decoding measure is only an indirect measure and does not directly illustrate the structure of the representational space and the orthogonality of object representations in VWM. They stated that to make a convincing case, a direct depiction of the representational space, together with the measurements of the representational angles, needs to be reported. Hence, I included these additional measures in the revised manuscript. But I agree with this reviewer that the angle measurements do not add much beyond the cross-decoding analysis: they reflect the same underlying representational structure.

> *3) Power is more than the number of subjects – it is a function of the number of subjects and trials within each subject. Just mentioning because this is a well-powered study but your justification only focuses on sample size.*

I agree with the reviewer here completely. This was again a point brought up in the last round of review where there was a concern that the sample size might have been insufficient. As such, extra texts were added to justify the sample size here.
